# Intermediate Filaments as Effectors of Cancer Development and Metastasis: A Focus on Keratins, Vimentin, and Nestin

**DOI:** 10.3390/cells8050497

**Published:** 2019-05-23

**Authors:** Pooja Sharma, Sarah Alsharif, Arwa Fallatah, Byung Min Chung

**Affiliations:** Departments of Biology, The Catholic University of America, 620 Michigan Ave NE, Washington, DC 20064, USA; 48sharma@cua.edu (P.S.); 47alsharif@cua.edu (S.A.); 84fallatah@cua.edu (A.F.)

**Keywords:** cancer, metastasis, hallmarks of cancer, intermediate filament, keratin, vimentin, nestin

## Abstract

Intermediate filament (IF) proteins make up the largest family of cytoskeletal proteins in metazoans, and are traditionally known for their roles in fostering structural integrity in cells and tissues. Remarkably, individual IF genes are tightly regulated in a fashion that reflects the type of tissue, its developmental and differentiation stages, and biological context. In cancer, IF proteins serve as diagnostic markers, as tumor cells partially retain their original signature expression of IF proteins. However, there are also characteristic alterations in IF gene expression and protein regulation. The use of high throughput analytics suggests that tumor-associated alterations in IF gene expression have prognostic value. Parallel research is also showing that IF proteins directly and significantly impact several key cellular properties, including proliferation, death, migration, and invasiveness, with a demonstrated impact on the development, progression, and characteristics of various tumors. In this review, we draw from recent studies focused on three IF proteins most associated with cancer (keratins, vimentin, and nestin) to highlight how several “hallmarks of cancer” described by Hanahan and Weinberg are impacted by IF proteins. The evidence already in hand establishes that IF proteins function beyond their classical roles as markers and serve as effectors of tumorigenesis.

## 1. Introduction

### 1.1. Intermediate Filament Family of Cytoskeletal Proteins

**Figure 1:**
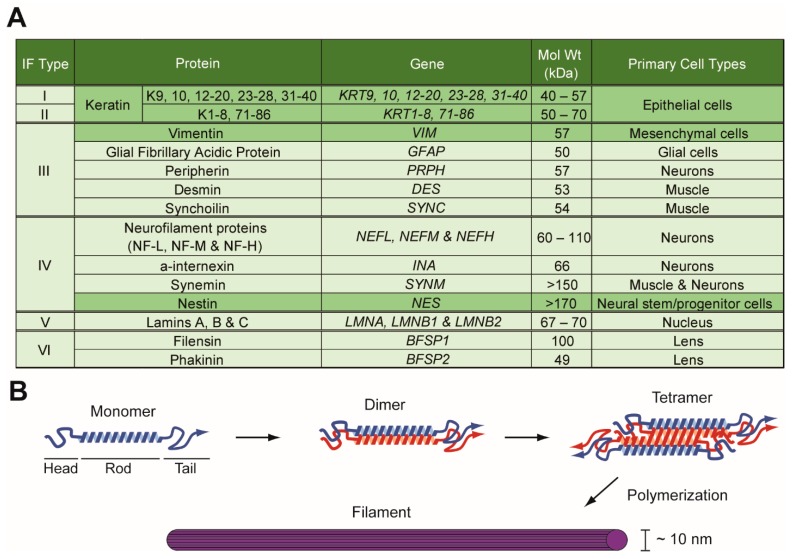
Intermediate Filament (IF) Family of Proteins. (**A**) List of IF proteins, along with their class types, gene names, molecular weights, and expression patterns. Proteins discussed in this review are highlighted in dark green. (**B**) Schematic representation of the domain structure and polymerization of IF proteins. IF proteins become either homo- or heterodimerized through coiled-coil interactions between rod domains of monomers. Then, two dimers assemble in an antiparallel manner to form a nonpolar tetramer. Further polymerization of tetramers result in unit-length filaments and ultimately a filament of about 10 nm in diameter. For keratins, heterodimerization occurs between one type I and one type II keratin. While vimentin can form filaments on its own, nestin requires other IF proteins to form filaments. An arrow on the tail domain indicates the C-terminal end of the protein.

The intermediate filament (IF) family of proteins is composed of 73 genes, making it the most diverse family of cytoskeletal proteins, which also includes microtubules and actin filaments [1]. The intermediate filaments received its name due to its 10 nm filament width being intermediate between those of 25 nm wide microtubules and 6 nm wide actin filaments. Each gene within the intermediate filament family is categorized into one of six major subtypes, based on primary amino acid sequence similarity, with expression occurring in nearly all eukaryotic cell types (Figure 1A). Despite the large number of genes in its family, any given cell in the body expresses only a subset of the family members. Thus, the expression of each member is tightly regulated in a tissue-, differentiation-, or context-dependent manner. Remarkably, all IF proteins share a similar structure in that a highly conserved central α-helical rod domain is flanked by more divergent head and tail domains (Figure 1B) [2]. It is the central rod domain that enables the self-assembly into oligomers and formation of 10 nm filaments [3].

As cytoskeletal proteins, IF proteins serve an important role in that they maintain critical structural integrity of cells and tissues. This is illustrated by mutations in IF genes, which result in abnormal filament formation and directly cause a wide range of rare human diseases that manifest in disrupted tissue integrity, such as skin blistering or abnormal muscle fiber formation [4]. Interestingly, studies involving IF function in the context of human diseases as well as normal tissue homeostasis have revealed that IF proteins serve multiple non-mechanical support functions [2,5,6]. It is now widely appreciated that IFs regulate various cellular processes ranging from cell migration to apoptosis and proliferation [2,7,8]. In addition to mutations observed in several diseases, as discussed above, an aberrant expression of IF proteins can also be observed in diseases such as alcoholic and nonalcoholic steatohepatitis [9] and psoriasis [10], where a change in the levels of IF proteins plays a critical role in altering cellular processes to facilitate disease progression [7,11]. In this review, we will discuss the functions of IF proteins in cancer, focusing on a subset of IFs that have been most widely studied in the context of cancer—keratins, vimentin, and nestin.

Keratins belong to a family of IF proteins that is expressed in all epithelial cells, where they provide crucial structural support upon mechanical and non-mechanical stresses. The 54 keratin genes with 28 type I and 26 type II sequences are regulated so that Type I and II keratins heterodimerize and undergo further polymerization to form filaments, leading to a requirement for pairwise regulation at the transcriptional and post-translational levels [5]. As it is for the IF family as a whole, not all keratins are expressed in a given cell. Rather, only a subset are expressed in a tissue-specific, differentiation-dependent, and developmentally-regulated manner [5,12]. Profile of keratin expression, therefore, allows classification of epithelial cells [13,14]. For example, K8, K18, and K19 are found in simple epithelial cells, whereas K5 and K14 are expressed in basal epithelial cells. Keratin filaments can be dynamically remodeled and can undergo reorganization upon various mechanical and non-mechanical stimuli to regulate a variety of cellular processes, including cell signaling and migration [5,7].

Vimentin is a 57 kDa type III IF protein that is expressed mainly in mesenchymal cell types, including fibroblasts, bone-marrow-derived blood cells, and endothelial cells (Figure 1A) [7,15]. As a major IF protein in mesenchymal cells, vimentin is critical for a number of cellular functions, including cell adhesion, migration, and signaling [16]. Most notably, in cancer, vimentin is most frequently used as a marker of mesenchymal cell types in epithelial-to-mesenchymal transition (EMT), a process that is critical to cancer metastasis [15,17].

Originally discovered in neuroepithelial stem cells [18], nestin is a class VI IF protein that continues to be used as a marker of neural stem and progenitor cells in the central nervous system but is also expressed in other tissue types, including endothelial cells (Figure 1A) [19]. With a long C-terminal end, nestin is a large protein at >170 kDa that cannot form filaments by itself but instead interacts with other IF proteins to form filaments. In cancer, nestin has been found to be expressed in cancer stem-like cells and poorly differentiated cancer cells [19,20,21].

### 1.2. IF and Cancer—IFs as Diagnostic and Prognostic Markers of Cancer

When normal cells transform to cancerous cells, original signature expressions of IF proteins are largely retained. As the vast majority of cancer cells originate from epithelial cells, staining of IFs, most notably keratins, has proven to be a useful tool for pathologists to identify tumor and cell types [14,22]. Using a series of monoclonal antibodies reported in the early 1980s, staining of keratins has been used to diagnose human tumors in a clinic [22]. Expression of keratins can also be used to classify cancer types; as in the case of non-small cell lung cancer (NSCLC), K17 is overexpressed in squamous carcinomas compared to the adenocarcinomas, whereas the reverse is true for K8 and K18 [14]. Clinical applications of keratins also involve detection of K8, K18, and K19 fragments from serum as diagnostic tools [23,24,25,26]. For these reasons, diagnosing cancer using anti-keratin antibodies is especially valuable for detecting and characterizing metastatic tumors of unknown origin [27].

A growing number of studies are showing that altered expression of a subset of IF proteins might contribute to patient prognoses [27]. This is most notable for vimentin, which is upregulated in cancer cells that have undergone EMT [15]. A number of keratins also exhibit altered expression in various tumors compared to the normal tissues [27]. While there are some keratins in certain cancer types that show a favorable prognosis when expressed at higher levels, many studies have found increased expressions of select keratins to correlate with more aggressive tumor types and worse patient prognosis [27]. For example, in breast cancer, downregulation of K18 is significantly correlated with advanced tumor stage and shorter patient survival [28], but studies examining K5, K14, and K17 have shown that overexpression of these proteins are associated with poor patient prognoses across various cancer types [29,30,31]. Also, altered levels of K19 are associated with poor survival in patients of various cancer types [27,32]. Mechanisms of change in IF gene expression in cancer settings involve transcriptional regulation [33,34] but recent findings also suggest that post-transcriptional [35,36], as well as post-translational [37], regulations are also involved. To our knowledge, there has been only one mutation associated with increased risk in cancer—K5 G138E in basal cell carcinomas [38]—so altered levels of IF proteins seemed to be the primary way they exert effects on cancer cells.

While correlations between altered IF expression and patient survival suggested that IF may play an active role in tumorigenesis, it was not until studies were performed using animal and cell culture models that this became clearer. In the following sections, the molecular mechanism involving these IF in cancer cells will be summarized. We will examine cancer hallmarks, described by Hanahan and Weinberg [39,40], to assess the impact of IF proteins towards these cellular processes that ultimately contribute to tumorigenesis. For these processes, we will focus mostly on keratins and encourage readers to read reviews on vimentin [15,41] and nestin [42,43] for those areas we do not fully cover.

## 2. Impacts of Intermediate Filament Proteins on Cancer Hallmarks

### 2.1. Sustaining Proliferative Signaling

**Figure 2:**
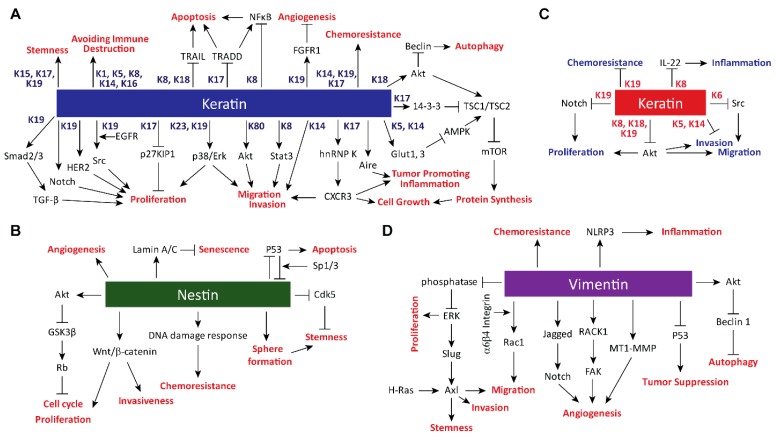
Signaling Pathways and Cellular Processes Regulated by Keratins, Vimentin, and Nestin. Proteins and cellular processes activated (arrows) or inhibited (bar-headed lines) by (**A**,**C**) keratins, (**B**) nestin, and (**D**) vimentin. (**A**) Keratins in blue are involved in either activation or inhibition of proteins in black, and upregulate pro-tumorigenic processes in red. For example, K19 enables activation of multiple signaling molecules, including HER2, Src, and Notch for cell proliferation. Alternatively, multiple keratins may converge on a same cellular process as illustrated by regulation of cell migration by K8, K14, K17, K19, and K80 through various signaling molecules, including STAT3, hnRNP K, p38, Erk, and Akt. Processes, such as chemoresistance, that are directly linked to keratins indicate lack of known mediators. (**B**) Downstream factors and pro-tumorigenic processes in red regulated by nestin. Some of the better-known roles of nestin include maintaining cell stemness through CDK5 inhibition. (**C**) Keratins in red inhibit proteins in black and downregulate pro-tumorigenic processes in blue. For example, K6 inhibits cell migration by inactivating Src. Interestingly, K19 activates Notch signaling pathway to promote cell proliferation in hepatocellular carcinomas but inhibits Notch signaling to suppress proliferation of breast cancer cells. Such a difference exemplifies context-dependent functions of keratins. (**D**) Downstream factors and pro-tumorigenic processes in red regulated by vimentin. Vimentin is upregulated in cells that have gone through the epithelial-to-mesenchymal transition, and it promotes invasion and migration via activation of Erk and Rac1 signaling pathways.

Phosphoinositide 3-kinase/Akt pathway is one of the major oncogenic signaling pathways activated in human cancers and regulates various cellular processes, including cell proliferation and migration [44,45]. Using keratinocytes, it has been shown that select keratins are required for the activation of Akt and its downstream signaling molecule mechanistic target of rapamycin (mTOR). Wound-inducible keratin K17 regulates Akt and mTOR signaling and protein synthesis through its interaction with 14-3-3σ, which relieves repression on Akt/mTOR signaling (Figure 2A) [46]. Alternatively, K5 and K14 regulate mTOR signaling by regulating GLUT1 and -3 upstream of AMP Kinase and Raptor (Figure 2A) [47]. Interestingly, NSCLC A549 cell line stably expressing nestin shRNA also showed decreased phosphorylation of Akt, as well as GSK3β (Figure 2B) [48], suggesting that different IFs may employ a similar effect towards the Akt signaling pathway.

Mouse models of cancer have supported the role of K17 in tumor progression. Transgenic mice with skin-specific overexpression of Gli2, an effector of Sonic hedgehog signaling pathways, develop K17-positive basal cell carcinoma-like skin lesions [49]. When compared to *Gli2^tg^* animals, *Gli2^tg^; Krt17*^−/−^ mice showed a delayed tumor onset (Table 1) [50], demonstrating that K17 plays an active role in tumor development. Similarly, a transgenic mouse model of squamous cell carcinoma with *HPV16^tg^* in skin further showed that K17 is required for the proper onset of tumors (Table 1) [51]. In addition to these skin tumor models, xenograft assays with nude mice injected with K17 shRNA expressing A673 or SK-N-MC Ewing Sarcoma cells [52] and SiHa and CaSki cervical cancer cells [53] also revealed K17 to be critical for aggressive tumor growth in vivo (Table 1). In contrast, K10, which is normally expressed in the superbasal layer of the skin epidermis, has been suggested to inhibit tumorigenesis. Forced expression of K10 in the basal layer of the epidermis resulted in impaired tumorigenesis upon exposure to chemical carcinogen 12-O-Tetradecanoylphorbol-13-acetate (TPA) (Table 1) [54].

In breast cancer, K19 was shown to be critical for the proper activation of signaling pathways involving receptor tyrosine kinase epidermal growth factor receptor (EGFR) family members. In SKBR3 breast cancer cells, K19 is required for Src activity downstream of EGFR to drive cell proliferation (Figure 2A) [68]. Consistent with this, another study showed that K19 binds to and stabilizes another EGFR family member, HER2, on the cell surface (Figure 2A) [56]. Accordingly, treating breast cancer cells with anti-K19 antibody decreased HER2 level, cell proliferation, and in vivo tumor formation in a xenograft mouse experiment (Table 1) [56]. Interestingly, however, a different study showed that depleting K19 using shRNA in SKBR3 cell line increased tumor formation in nude mice (Table 1) [57], complicating the role of K19 in breast cancer.

K19 has also been shown to regulate the Notch signaling pathway for cell proliferation, albeit with different effects on different cancer cell lines. Huh7 hepatocellular carcinoma cells expressing K19 shRNA showed decreased cell proliferation along with decreased levels of NOTCH1, JAG1, DTX1, and TGFBR1, along with phosphorylated SMAD2 and SMAD3 (Figure 2A) [69]. However, knockdown of K19 in MDA-MB-231 and MCF7 breast cancer cells led to increased proliferation by upregulating the Notch signaling pathway (Figure 2C) [70]. Different roles of K19 in cell proliferation may be attributed to a biphasic effect, where K19 engages different signaling pathways depending on different contexts [56].

Other cell proliferative signaling pathways have also been shown to be dependent on IF proteins for activation in cancer cells. Depletion of K23 in colorectal cancer inhibited the proliferation and migration by decreasing activation of downstream targets of EGFR, p38, and Erk (Figure 2A) [71]. Likewise, vimentin also regulates the proliferation of fibroblasts through Erk signaling (Figure 2D) [72], suggesting that the regulation of Erk signaling may be conserved in different IF members.

For nestin, the general consensus is that it promotes cancer, as multiple studies showed that depleting nestin expression resulted in decreased xenograft tumor formation in vivo. Xenograft mouse model studies showed that 5-8F nasopharyngeal carcinoma cells [63], hepatocellular carcinoma cell line Huh7 [64], and human glioblastoma cell line A172 [65] expressing nestin shRNA yielded decreased tumor volume compared to those from control cells (Table 1). Similarly, formation of liver cancer by co-injection of transposons encoding tumor oncogene YAP and p53 shRNA into mice was abrogated when nestin shRNA was co-injected [64] and administering nestin siRNA by subcutaneous injection decreased tumor volume of human pancreatic cancer cell line KLM-1 in nude mice (Table 1) [67]. All of these data suggest that targeting nestin may be a viable therapeutic option in cancer. As for the underlying basis of nestin on tumor growth, Wnt signaling seems to be required, as depleting nestin via siRNA transfection downregulated activation of Wnt/β-catenin, which is critical for the proliferation of human breast cancer stem cells (Figure 2B) [73]. Overall, these data collectively demonstrate that at least a subset of keratins along with nestin and vimentin can regulate cell proliferative signaling.

### 2.2. Evading Growth Suppressors

In the context of cancer, IFs can enhance tumorigenesis by inhibiting tumor suppressors. For example, K17 interacts with a tumor suppressor and cell cycle inhibitor p27KIP1 and promotes its nuclear export for its degradation in human cervical cancer cell lines [53], and this K17-dependent regulation of p27KIP1 enhances cell proliferation (Figure 2A). This study is one of several examples of the ability of IF proteins to regulate protein function by affecting its subcellular localization [2,74,75,76,77]. For example, expression of vimentin has been shown to be correlated with cytoplasmic localization of p53 in human primary glioblastomas (Figure 2D) [78], raising a possibility that vimentin interacts with p53 to regulate its localization. Interestingly, another tumor suppressor, NF1 protein, which regulates Ras activity, has been shown to associate with K14 in the basal epidermal layer during development in skin [79], suggesting that K14 may regulate the function of this tumor suppressor.

Given the pro-tumorigenic functions of select IF proteins, levels of their expression seem to be kept in check by tumor suppressors in normal cells. For an example, nestin expression is inhibited by p53 in an Sp1/3 transcriptional factor-dependent manner (Figure 2B) [64]. In turn, formation of liver cancer through depletion of p53 levels was dependent on nestin expression (Figure 2B) [64]. Likewise, in many human hepatocellular carcinomas or cholangiocarcinomas, nestin expression is correlated with loss of p53 expression, where it is associated with poor patient survival [64]. These findings suggest that nestin plays a key tumorigenic role in cells that have escaped the control of tumor suppressors. Similarly, chemical induction of skin tumors involving TPA in mice devoid of tumor suppressor SIRT2 also increased the expression of K19 [80], which marks stem cells in skin and cancer stem cells in squamous cell carcinoma [81,82]. It is likely that additional pro-tumorigenic IF proteins are also regulated by tumor suppressors, and elevated levels of these IF proteins may play an integral role in the development of cancers where tumor suppressors are absent.

### 2.3. Resisting Cell Death

Resisting cell death is an important feature of tumor cells, allowing them to withstand a myriad of challenges, including chemotherapy, in order to proliferate. Clinical correlations between altered IF protein expression and resistance against chemotherapy and radiation therapy have been observed in several cancer types [27], and emerging data confirm the impact of IF proteins on resisting cell death. This is particularly true for those IF proteins, such as nestin, that are critical for cancer stem cell maintenance, as cancer stem cells play key roles in resistance to conventional chemotherapy [83,84]. In glioma, nestin-positive cells that mark a stem-cell-like population allow tumor cells to survive and propagate upon exposure to a chemotherapeutic agent [85]. Indeed, nestin is required to properly induce the DNA damage response to prevent cell death. Depleting nestin using shRNA in 5-8F nasopharyngeal carcinoma cells resulted in increased spontaneous DNA damage accumulation and delayed doxorubicin-induced DNA damage repair (Figure 2B) [63]. Consistently, nestin knockdown cells also displayed increased sensitivity to γ-irradiation in a xenograft assay [63]. Higher expression of vimentin has also been correlated with chemoresistance in various cancer types [86,87]. Therefore, targeting IF proteins in tumor may benefit patients clinically.

Besides nestin and vimentin, keratins also play key roles in resisting cell death. For an example, using gemcitabine and cisplatin, the standard chemotherapy regimen for advanced bladder urothelial carcinomas, on xenograft tumors in mice demonstrated that chemotherapy-resistant bladder cancer stem cells express K14 (Figure 2A) [88]. Interestingly, a series of findings showed conflicting roles of K19 in resistance to cell death. For instance, K19-positive cancer initiating cells are resistant to radiation in a mouse model of colon cancer (Figure 2A) [89]. However, knockdown of K19 in MDA-MB-231 and MCF7 cells increased resistance to doxorubicin (Figure 2C) [70], and overexpression of K19 in BT549 breast cancer cell line resulted in decreased sensitivity towards cisplatin and doxorubicin (Figure 2C) [90]. It is most likely that these results arose from context-dependent and tissue-specific functions of keratins.

Knockdown of K17 in cervical cancer cell lines resulted in increased sensitivity to cisplatin, suggesting that K17 also drives the resistance to chemotherapy (Figure 2A) [53]. Interestingly in skin epidermis during the hair follicle cycle, K17 modulates TNFα signaling and NFκB activity through its interaction with an adaptor protein TRADD to suppress apoptosis (Figure 2A) [91], thus, a similar mechanism may take place in cancer settings. Similarly, downregulation of K8 altered TMS1-NF-κB signaling cascade, leading to increased apoptosis and significantly reduced tumorigenic potential of skin squamous carcinoma cells (Figure 2A) [92]. K8 and K18 also function in the liver to help hepatocytes cope with mechanical and nonmechanical stress that can result in apoptosis and necrosis [93,94], and a recent study by Bozza et al. also showed that K8 and K18 protect breast cancer cells from TRAIL-induced apoptosis by downregulating death receptor expression (Figure 2A) [95].

One of the mechanisms a cell utilizes to resist cell death is autophagy, a process in which a cell digests its own proteins and organelles to sustain its metabolism [96]. IF proteins play a central role in a crosstalk between oncogenic proteins and autophagy proteins to balance the growth potential of tumor cells. Both vimentin and K18 interact with tumor suppressor Beclin through an adaptor protein 14-3-3 by Akt activation (Figure 2A,D) [77]. This complex formation involving IF proteins is critical for inhibiting autophagy while facilitating tumor cell growth, as shRNA-mediated suppression of vimentin expression increased autophagy while decreasing Akt-mediated tumor cell growth in rat2 fibroblasts [77]. As K5 and K14 [47], K17 [46], and nestin [48] have been shown to regulate the Akt/mTOR pathway (Figure 2A), which plays a critical role in autophagy [97,98], it is likely that several IF proteins serve as regulators of autophagy in various tissues.

### 2.4. Enabling Replicative Immortality

For many years, select IF proteins, most notably nestin, have been used as markers of cancer stem cells in various cancer types. In breast cancer, for example, nestin expression in tumors of human patients was shown to be significantly associated with aggressive cancer type with stem-like features [99]. The role of nestin on tumorigenesis and stemness of cancer cells has previously been reviewed by others [19,20,100]. Findings since then have provided support for the role of nestin as a critical element in stemness of cancer cells. Nestin expression is correlated with tumor size, lymph node metastasis, and poor survival in NSCLC patients [21]. Reduced expression of nestin by shRNA in NSCLC cell line H1975 [21], pancreatic cancer cell line PANC, and glioblastoma cell line A172 [65] showed decreased sphere formation in vitro, which measures the self-renewal capacity of cancer cells (Figure 2B). Similarly, CD44^high^/CD24^low^ breast cancer stem cells isolated from human tissues transfected with nestin siRNA showed reduced sphere formation, cell cycle arrest at G2/M, and increased apoptosis (Figure 2B) [73]. Reduced expression of nestin through shRNA in NSCLC cell lines A549 and A460 also resulted in cell cycle arrest at G1 and decreased cell proliferation (Figure 2B) [48].

While the molecular detail of how nestin exerts its effect on stem cell maintenance remains largely unknown, at least one of the ways is through Cdk5. Nestin binds and inhibits Cdk5 activation to inhibit differentiation of muscle cells for stem cell maintenance (Figure 2B) [101,102]. Furthermore, a recent finding showed that nestin-dependent regulation of Cdk5 occurs inside the nucleus, where nestin interacts with lamin A/C to maintain nuclear integrity and protect tumor cells from senescence (Figure 2B) [103].

Select keratins have also been used to identify cancer stem cells. Basal and squamous cell carcinomas in skin originate from K15-positive stem cells of the follicular bulge [104,105], which suggests that K15 may have a role in cancer stem cell maintenance (Figure 2A). In cervical epithelia, K17 is a marker of the reserve cell, a stem-like cell that gives rise to endo- and ectocervical tissue compartments [106], and loss of K17 suppresses estrogen- and HPV16^tg^-induced cervical tumorigenesis in mice (Table 1) [107]. Consistent with this, K17 enhances cancer stem cell-like properties in cervical cancer. Silencing K17 expression suppressed cancer stem cell-like properties, and overexpression of K17 promoted metastasis (Figure 2A) [108]. In breast cancer, K19 may be involved in stem cell maintenance for cancer metastasis. Introducing a set of oncogenes, including mutant H-Ras, mutant p53, and either EGFR or ErbB2, to mammary stem and progenitor cell types demonstrated that K19-positive progenitor cells formed tumors with shorter latency and displayed higher metastatic potential in xenograft assay than K19-negative progenitor cells (Figure 2A) [109]. Of note, K19 is also a stem cell marker in several normal tissue types [84,89,110]. It is likely that depending on cancer or cell types, different IF proteins may play a role in maintaining stem cell properties and allowing cancer cells to proliferate.

Vimentin also seems to have a role in regulating stemness of cancer cells. Depleting vimentin expression through shRNA resulted in reduced population of breast cancer stem cells, as measured by high expression of aldehyde dehydrogenase (Figure 2D) [111], suggesting that vimentin is required for the maintenance of breast cancer stem cells. Interestingly, multiple studies have shown that cancer stem cells display traits of cells that have undergone EMT [83,84,112]. For example, EMT induction by snail or twist overexpression promoted the generation of cancer stem cells in human mammary epithelial cells, and markers of mesenchymal cells, including vimentin, was high in CD44^high^/CD24^low^ stem cells of breast cancer [113]. While it is unclear how vimentin promotes cancer cell stemness, one possible way may involve promoting Erk-mediated phosphorylation of slug for EMT initiation [76].

### 2.5. Inducing Angiogenesis

Angiogenesis is critical for tumors to receive nutrients and metastasize to distal organs. IF proteins, most notably nestin and vimentin present in endothelial cells, have been implicated in angiogenesis. Nestin is highly associated with angiogenesis, as it is expressed by newly formed blood vessels during embryogenesis and in various tumor tissues, including prostate and pancreatic cancers (Figure 2B) [114]. Not surprisingly, depleting nestin expression via siRNA resulted in decreased cell growth of vascular endothelial cell lines along with decreased subcutaneous human pancreatic cancer cell growth in nude mice (Table 1) [67].

In the case of vimentin, it is required for cell sprouting in endothelial cells. Vimentin was required for the membrane localization and proper activation of MT1-MMP, which in turn is required for the endothelial sprouting (Figure 2D) [115]. Similarly, human umbilical vein endothelial cells expressing vimentin shRNA showed decreased focal adhesion kinase expression, which forms a complex with vimentin and RACK1 during endothelial cell invasion for cell sprouting (Figure 2D) [116]. Recently, Antfolk et. al. showed that vimentin regulates Notch ligand signaling activities for angiogenesis, as vimentin binds to the proangiogenic Jagged ligands and regulates their endocytosis (Figure 2D) [117]. While the role of keratins in angiogenesis is very limited, a potential role of K19 in hepatocellular carcinoma (HCC) angiogenesis was observed. The number of blood vessels around cancer foci was significantly higher in K19-positive HCC specimens than in K19-negative HCC specimens and expression of angiogenesis-related genes, such as VASH1, FGFR1, and VASH2, was altered in K19-depleted HCC cell lines (Figure 2A) [118]. These studies show that IF proteins regulate multiple protein machineries involved in angiogenesis.

### 2.6. Activating Invasion and Metastasis

The requirement of IFs on metastasis occurs at two levels, which are not mutually exclusive and may in fact be intricately linked. First, as polymerized filaments, IF proteins provide mechanical elements that enable cells to invade and migrate through their surrounding tissues during initial stages of tumor metastasis. This element of IF proteins is likely to involve other cytoskeletal proteins, as mechanical properties and dynamics of intermediate filaments are linked to those of microtubules and actin filaments to propel cells forward [119,120,121,122]. Second, IF proteins have been found to regulate signaling pathways in cell migration through their interacting partners. Among IFs, vimentin and nestin are positive regulators of cell migration and invasion [121,123,124,125], due in part to their roles in signaling events during EMT and stem cell maintenance, respectively, as mentioned in previous sections. In contrast, different keratins have been shown to regulate migration differently. In fact, different studies have reported opposing roles of same keratin proteins in certain cases. This is likely due to the context-dependent functions of keratins and presence of other keratins in the system. As involvement of IFs on cell migration, invasion, and metastasis has been reviewed [7,126], we will briefly summarize what was known and highlight the latest developments involving IFs on cell invasion and metastasis.

Vimentin has been shown to be required for tumor metastasis in a number of animal studies. Interestingly, however, an earlier study of a teratocarcinoma model using injection of vimentin null embryonic stem cells showed that vimentin had no effect on tumor growth (Table 1) [60]. Still, subsequent studies using cancer cells with additional genetic alterations revealed the requirement of vimentin in metastasis. For example, a transgenic mouse model of lung cancer with *Kras*^G12D^, *Lkb1*^fl/fl^ showed that although primary tumor growth was unaffected, metastasis was inhibited by the absence of vimentin (Table 1) [59]. Furthermore, injection of MVT-1 mammary tumor cells with vimentin shRNA into a pre-diabetic mouse model showed the requirement of vimentin for metastasis in a pre-diabetic condition (Table 1) [61]. In pancreatic cancer, transfection of nestin shRNA in PANC cells caused decreased metastasis in immunodeficient mice upon intrasplenic injection (Table 1) [66], suggesting that nestin may also be involved in metastasis.

Engaging in multiple signaling pathways helps vimentin to promote cell motility. Vimentin is required for the expression of receptor tyrosine kinase Axl downstream of Slug or oncogenic H-Ras (Figure 2D) [127]. In addition, vimentin, along with α6β4 integrin, serves as a signaling hub in A549 cells to activate Rac1 signaling for cell migration (Figure 2D) [128]. A study by Virtakoivu et al. also showed that vimentin facilitates cell migration by directly associating with Erk and recruiting Slug for the Erk-mediated phosphorylation (Figure 2D) [76]. In line with the cell migration-promoting role of vimentin through its interactions with signaling proteins, hyperphosphorylation of Akt target site on vimentin, S39, has been shown to enhance migration and invasion of soft tissue sarcoma, and metastasis in a xenograft model (Table 1) [62].

As for keratins, they appear to affect cell motility in a context-dependent manner, as mentioned above. Loss of all keratins through genetic ablation decreased keratinocyte stiffness and increases cell invasiveness [129]. Interestingly, re-expression of basal keratins K5 and K14 reversed these phenotypes [129], indicating that K5 and K14 inhibit cell invasion (Figure 2C). However, in breast cancer, K14 expression in leader cells is required for tumor cells to collectively invade their surrounding microenvironment for metastasis [55], and a similar observation was made using salivary adenoid cystic carcinoma cells in a 3D culture assay (Figure 2A) [130].

Opposing functions in invasion and migration have also been observed for K19 [57,90,131], despite the positive correlation between increased K19 expression and metastasis in cancer patients [69,132,133,134,135,136]. Knockdown of K19 using shRNA in Huh7 cells showed decreased invasion in a transwell assay compared to those expressing control shRNA (Figure 2A) [69]. This, at least in part, involves platelet-derived growth factor receptor α (PDGFRα)-dependent expression of laminin beta 1 and α2β1 integrin receptor signaling pathway (Figure 2A) [137]. However, overexpressing K19 in K19-negative BT549 breast cancer cell line resulted in decreased cell migration in an in vitro wound healing assay [90]. Similarly, overexpression of K19 in oral squamous cell carcinoma lines also resulted in decreased cell migration and invasion [131]. The inhibitory role of K19 on cell migration was also demonstrated when silencing K19 in BT474 and SKBR3 breast cancer cells using shRNA, which resulted in increased cell proliferation, migration, invasion, and survival through upregulation of Akt signaling pathway (Figure 2C) [57]. In contrast, a recent study showed activation of Akt signaling for cell migration and invasion by a type II keratin K80, which is upregulated in human colorectal carcinomas and interacted with protein kinase, DNA-activated, catalytic polypeptide (PRKDC) for subsequent expression of EMT markers (Figure 2A) [138].

As for keratins in simple epithelial cells, several studies have shown previously that K8 and K18 negatively regulate invasion and migration of multiple cancer cell lines [28,139,140,141]. This is, in part, by inhibiting Akt and NFκB activities (Figure 2C) [140]. However, knockdown of K8 expression in a renal cancer cell line showed that K8 is required for cancer metastasis through IL-11 expression and STAT3 activation (Figure 2A) [142], revealing a metastasis-promoting role of K8 and providing yet another example of the highly context-dependent nature of the keratin function.

Phosphorylation of keratin may also play a role in metastasis. During tissue injury or stress, K8 becomes phosphorylated at the p38 target site, S73, and Erk target site S431 [143,144]. K8 dephosphorylation on S73 correlated with increased tumor size and lymph node metastasis along with worse patient prognosis among human oral squamous cell carcinoma tissues (Table 1) [58]. Consistent with this clinical observation, immunodeficient mice injected with AW13516 human oral squamous cell carcinoma cells expressing K8 mutants S73A or S431A displayed significantly higher tumor volume compared to those injected with K8 wild type clones (Table 1) [58]. As IF proteins readily undergo multiple post-translational modifications that are critical for various functions of IF proteins [6,7,144], it is likely that additional post-translational modifications involving other IF proteins affect tumor growth and metastasis. It will be important to delineate kinases and phosphatases regulating IF protein phosphorylation and to determine the mechanistic detail of how phosphorylated IF proteins impact tumor progression.

Studies on K17 showed a novel mechanism of IF-dependent cell migration that involves gene expression and chemokine secretion. K17 is required for the cell invasion of A431 skin cancer cells, as cells expressing K17 shRNA displayed decreased invasion towards EGF in a transwell invasion assay [74]. Interestingly, K17 exerted a paracrine effect on cell migration, as it was required for the proper expression of CXCR3 ligands, chemokines CXCL9, CXCL10, and CXCL11 (Figure 2A) [74]. Indeed, CXCR3-expressing A431 cells were less invasive towards the conditioned medium from K17 knockdown cells compared to the conditioned medium from the CXCR3 ligand-secreting control cells, indicating that K17 is required for the recruitment of CXCR3-positive cells via chemotaxis [74]. In addition, K17 has been shown to be inside the nucleus, where it regulates transcription of NFκB-dependent genes [51]. These events are likely to contribute to the recruitment of inflammatory cells to microenvironments around K17-expressing tumors [50,51].

### 2.7. Tumor-Promoting Inflammation

The recruitment and infiltration of effector innate and adaptive immune cells to sites of tumor growth has long been documented. The inflammatory uptick during tumorigenesis has classically been associated with the targeting of tumor cell antigens by immune cell recognition to lead to elimination by the immune system. However, elevated or prolonged bouts of inflammation may also foster a tissue environment conducive to tumor growth and progression. IF protein expression often becomes altered under inflamed tissue settings relative to homeostatic controls; such is the case for keratins in tumor-derived epithelia, as well as for vimentin and nestin in immune cell populations.

K17 regulates the expression of several key pro-inflammatory T_h_1- and T_h_17-type cytokines, including CXCR3 ligands [50,51,74], which can influence both epithelial cell motility (discussed above) and the recruitment of effector immune cells to sites of tumor onset. This ability of K17 to regulate gene expression is currently attributed to two key functions: (i) K17 interacts with the RNA binding protein hnRNP K, which associates with the mRNA transcript of CXCR3 ligands to regulate their expression (Figure 2A) [74]; and (ii) K17 is capable of localizing to chromatin-sparse (and presumably transcriptionally active) regions within the nucleus, where it can co-localize with the transcriptional regulator AIRE and associate with promoter regions of pro-inflammatory transcripts found to be K17-dependent for their expression (Figure 2A) [51]. Thus, K17 influences inflammatory gene expression at both the transcriptional and post-transcriptional levels. Future work will determine whether and how these events may be coordinated.

On the other hand, keratins may suppress inflammation required for tumorigenesis. The best understood work comes from work on K8 in the colon. K8 loss, as well as K8 mutation, was first correlated with colorectal hyperplasia and inflammation in 1994 [145], and has since been linked to T_h_2-driven colitis and inflammatory bowel disease [146,147]. K8^−/−^ mice do not spontaneously develop colorectal carcinomas; rather, they require a genetic “hit” (e.g., a mutation in the tumor suppressor *APC* gene) to prime the system. When crossed with a well-established model for colorectal cancer, the APC^Min/+^ mouse, K8^−/−^ mice robustly develop colorectal carcinomas, while K8^+/−^ littermates do not [146]. The reported mechanism behind this involves an association with the inflammasome and a robust activation of the Il-22 signaling pathway, specifically in the K8^−/−^ setting (Figure 2C) [146].

A number of reports indicate that vimentin and nestin expression become upregulated in inflammatory tissue settings. Elevated vimentin expression is correlated with inflammation in lung tissue injury models [148]. Mechanistically, vimentin can physically bind to, and activate, the NLRP3 inflammasome, a potent stimulator of the innate immune response, in alveolar macrophages (Figure 2D) [148]. Whether this ability of vimentin applies to tumor growth remains to be determined. For nestin, too, inducible upregulation is associated with a number of neurological diseases, including gliomas and astrocytomas [149,150]. However, little is known about how nestin directly contributes to this process mechanistically. Yet, nestin has been found to occur in nuclei of cell lines derived from neurological tumors [42], raising the possibility of a direct role in gene expression, as is the case for K17.

It is clear that alterations in the expression patterns of IF proteins are correlated with inflammation and tumor growth. The extent to which these altered expression patterns directly influence inflammatory gene expression and immune cell recruitment to tumor tissue environments have only recently emerged. Given the tissue- and context-dependent expression patterns for IF proteins, it appears likely that additional IF proteins (particularly the large family of keratins that can be robustly altered in epithelial-derived tumors) play a direct role in modifying inflammatory microenvironments.

### 2.8. Avoiding Immune Destruction

It is now established that cancer cells are capable of acquiring the ability to escape the immune response [25]. Examples of this include the avoidance of immune detection, alteration of immunogenicity, and the creation of an immunosuppressive microenvironment. Studies utilizing knockout mice for K1, K5, K8, and K16 all indicate an upregulated inflammatory profile in the skin (K1 [151], K5 [152], and K16 [153]) and liver (K8 [146]), which are linked to disease states (Figure 2A). For instance, K1 or K16 loss leads to a defective skin barrier and upregulation of the innate immune response [151,153]. In response to stress, K8 null mice exhibit a robust hepatitis phenotype [146]. Whether these settings contribute to the initiation or progression of cancer and afford cancer cells the ability to avoid the immune response remain to be determined. In this regard, a positive correlation was observed in basal bladder cancer cells between the expression of K14 and inflammatory mediators which promote immune suppression and tumor growth (Figure 2A) [154]. Thus, with the emergence of immunotherapy as a promising therapeutic option for cancer patients, it will be important to determine the mechanistic role of IF proteins on escaping the immune response to ensure treatment efficacy.

## 3. Conclusions

As summarized, IF proteins take on an active role in cancer development, progression, and metastasis. Despite differences in expression pattern and some of the cellular functions, there are many similarities between IF proteins, and the knowledge gained from one IF protein could very well be applied to other IF proteins. For instance, pro-tumorigenic IF proteins vimentin, nestin, and a subset of keratins, including K17, share a common trait in that all these proteins become induced upon wounding and tissue injury, when cells undergo hyper-proliferation [155,156].

Going forward, it will be important to determine how different intermediate filament proteins present in a given tumor coordinate their functions to affect tumor progression. Not only are several keratins present in the same tumors, but in certain cancer types, vimentin is co-expressed with keratins [13,14]. While different intermediate filament proteins, such as vimentin and keratins, assemble into separate filament networks with different dynamics [157], these two intermediate filament networks interact and are involved in a crosstalk [158], which plays a critical role in cell migration [27,159]. Due to the context-dependent expression and roles of intermediate filament proteins, deciphering how various intermediate filament proteins cooperate or antagonize each other’s functions would have valuable prognostic value for cancer patients.

Given the clinical correlation and the role of IF proteins in tumor progression, therapeutic reagents against IFs and therapeutic strategies against its associated signaling network may benefit cancer patients. Indeed, withaferin-A, a naturally derived bioactive compound that targets vimentin and induces vimentin cleavage, suppressed tumor growth and metastasis in various mouse models [160]. However, withaferin-A is the only small molecule that inhibits IF structure and function, and it targets several other cellular components [161]. Thus, it will be important to develop IF protein-specific small molecules to modulate their functions in cancer. Developing such a reagent, along with identification of the mechanisms underlying IF-mediated tumor progression, will be invaluable for cancer patients clinically.

## Figures and Tables

**Table 1:** Impact of IF genes on tumor growth and metastasis of mouse models. List of recent studies using transgenic or xenograft mouse models to implicate select keratins, vimentin, and nestin on growth or inhibition of tumors. Information about the mouse model, method of gene expression alteration, effect on tumor, and reference are shown. For transgenic mouse models, relevant tumor types along with promoters and transgenes or transposons used to drive tumor growth are shown. For xenograft models, relevant tumor types along with cell lines injected for tumor formation are shown. Outcomes upon IF gene alterations show changes in tumor growth and metastasis as results of IF gene mutations or expression or introduction of a blocking antibody. Each outcome was compared to that of the control setting, where IF protein remained intact.

IF Protein	Mouse Model	IF Gene Alteration	Outcome upon IF Gene Alteration	Ref.
K10	Transgenic-Skin carcinoma	overexpression of K10	Decreased tumor growth	[54]
	*bovineK5-hK10* injected			
K14	Xenograft-Breast carcinoma	K14 shRNA	Decreased metastasis	[55]
	*MMTV-PyMT* tumor cells injected			
K17	Transgenic-Skin carcinoma	*Krt17* ^−/−^	Decreased tumor growth	[50]
	*K5-Gli2tg*			
K17	Transgenic-Skin carcinoma	*Krt17* ^−/−^	Decreased tumor growth	[51]
	*K14-HPV16tg*			
K17	Xenograft-Ewing Sarcoma	K17 shRNA	Decreased tumor growth	[52]
	A673 and SK-N-MC cell lines injected			
K17	Xenograft-Cervical carcinoma	K17 shRNA	Decreased tumor growth	[53]
	SiHa and CaSki cell lines injected			
K19	Xenograft-Breast carcinoma	K19 antibody injected to block K19	Decreased tumor growth	[56]
	KPL-4 cell line injected			
K19	Xenograft-Breast carcinoma	K19 shRNA	Increased tumor growth	[57]
	SKBR3 cell line injected			
K8	Xenograft-Oral cavity squamous cell carcinoma	overexpression of KRT8 WT, S73A or S431A	K8 S73A and S431A increased tumor growth compared to WT	[58]
	AW13516 cell line injected			
Vimentin	Transgenic-Lung adenocarcinoma	*Vim* ^−/−^	Decreased metastasis	[59]
	*LSL-Kras^G12D^/Lkb1^fl/fl^*			
Vimentin	Xenograft-Teratocarcinoma	*Vim* ^−/−^	No change	[60]
	Embryonic stem cells injected			
Vimentin	Xenograft-*MCK–KR–hIGF-IR*	Vimentin shRNA	Decreased metastasis	[61]
	MVT-1 cell line injected			
Vimentin	Xenograft-Leiomyosarcoma	overexpression of VIM S39A or S29D	Vim S39D increased tumor growth and metastasis	[62]
	SKLMS1 cell line injected			
Nestin	Xenograft-Nasopharyngeal carcinoma	Nestin shRNA	Decreased tumor growth	[63]
	5-8F cell line injected			
Nestin	Xenograft-Hepatocellular carcinoma	Nestin shRNA	Decreased tumor growth	[64]
	Huh7 cell line injected			
Nestin	Transgenic-Hepatocellular carcinoma	Nestin shRNA	Decreased tumor growth	[64]
	Transposons encoding YAP and p53 shRNA injected			
Nestin	Xenograft-Glioblastoma	Nestin shRNA	Decreased tumor growth	[65]
	A172 cell line injected			
Nestin	Xenograft-Pancreatic carcinoma	Nestin shRNA	Decreased metastasis	[66]
	PANC cell line injected			
Nestin	Xenograft-Pancreatic carcinoma	Nestin shRNA	Decreased tumor growth	[67]
	KLM-1 cell line injected			
